# 171. A Multicenter Analysis of Inpatient Antibiotic Use During the 2015-2019 Influenza Season in the US: Untapped Opportunities for Antimicrobial Stewardship

**DOI:** 10.1093/ofid/ofab466.373

**Published:** 2021-12-04

**Authors:** Amine Amiche, Heidi Kabler, Janet Weeks, Kalvin Yu, Vikas Gupta

**Affiliations:** 1 Sanofi Pasteur, Dubai, Dubai, United Arab Emirates; 2 Becton, Dickinson and Company, Franklin Lakes, New Jersey

## Abstract

**Background:**

Inappropriate antibiotic (AB) use for viral respiratory illnesses remains widespread in the United States (US) with strong seasonal fluctuations. In contrast to outpatient AB use, the seasonality inpatient AB utilization (IAU) and its correlation with the influenza season are not well understood. We sought to describe trends, seasonality, and the association between IAU use and the 2015-2019 influenza seasons.

**Methods:**

We used the *BD Insights Research Database* (Franklin Lakes, NJ USA) to identify IAU that were prescribed in patients >17 years old from up to 236 US acute care facilities from July 2015 to December 2019. We included the following AB categories: extended spectrum cephalosporins (ESCs), macrolides, β-lactam inhibitor combination (BLIC), fluoroquinolones, carbapenems, glycopeptides, lipopeptide, tetracyclines, and others. We defined IAU use as days of therapy (DOT) per 1000 patient days present. We used influenza laboratory data to identify facility-level positivity ratio per 100 tests. We used random effect models to estimate IAU: 1) trends overtime, 2) seasonality, and 3) association with influenza positivity rate.

**Results:**

For IAU from 2015 to 2019, BLICs, ESCs, and glycopeptides were the most used [average 91, 107, and 96 DOT/1000 days presents, respectively]. Visually, we observed strong seasonality that matches the influenza season for macrolide, ESC, and quinolone use (See Figure). Unadjusted bivariate results showed ascending trends over time for BLICs [β= 3.8, p= .003], ESCs [β= 11.0, p= .005], and macrolides [β=1.5, p= .005]. Unadjusted bivariate results showed descending trends with quinolones [β= -10.9, p< .001] and others [β= -2.060, p< .001]. In the adjusted analysis, increased influenza positivity rate was associated with use of ESCs, glycopeptides, lipopeptides, macrolides, fluoroquinolone, and tetracyclines (see Table). No correlation was observed with BLICs, carbapenems, lipopeptides, and Others.

IAU (DOT/1000 days presents) and Flu Rate (% Positive) Trends Over Time

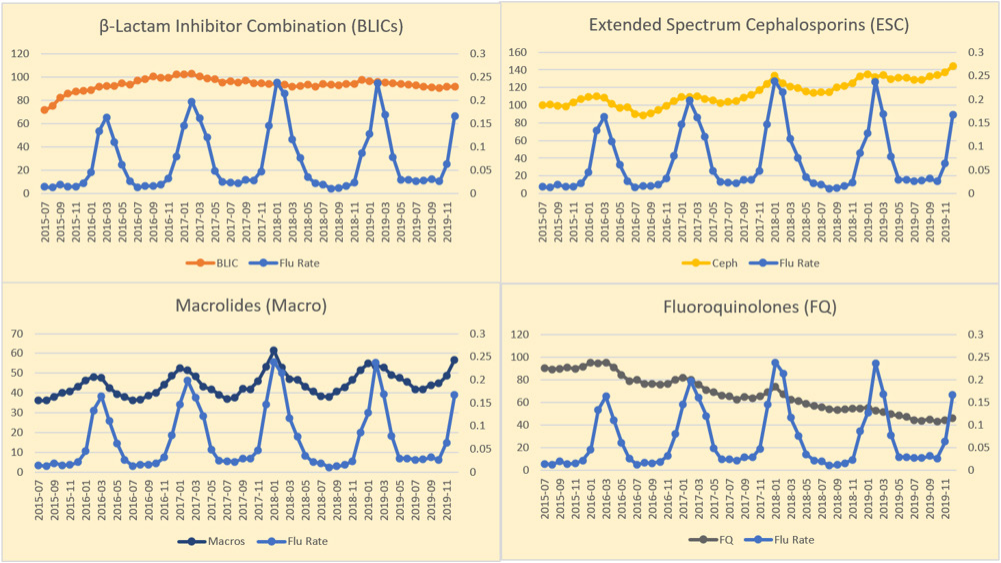

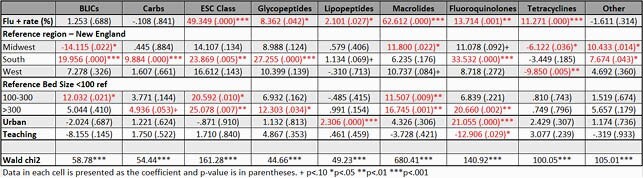

**Conclusion:**

Our study shows that IAU is on the rise for the ESC and BLIC classes. ESC and macrolide use was strongly correlated with influenza season. Monitoring influenza signals may provide more insights that can inform the interpretation of IAU trends and be incorporated into antimicrobial stewardship programs.

**Disclosures:**

**Amine Amiche, PhD**, **Sanofi** (Employee, Shareholder) **Heidi Kabler, MD**, **Sanofi Pasteur** (Employee) **Janet Weeks, PhD**, **Becton, Dickinson and Company** (Employee) **Kalvin Yu, MD**, **BD** (Employee) **Vikas Gupta, PharmD, BCPS**, **Becton, Dickinson and Company** (Employee, Shareholder)

